# Forgetting Alcohol: A Double-Blind, Randomized Controlled Trial Investigating Memory Inhibition Training in Young Binge Drinkers

**DOI:** 10.3389/fnins.2022.914213

**Published:** 2022-06-29

**Authors:** Natália Almeida-Antunes, Margarida Vasconcelos, Alberto Crego, Rui Rodrigues, Adriana Sampaio, Eduardo López-Caneda

**Affiliations:** Psychological Neuroscience Laboratory, Psychology Research Center, University of Minho, Braga, Portugal

**Keywords:** alcohol, craving, binge drinking, memory inhibition, randomized controlled trial, cognitive training, electroencephalograpy (EEG), transcranial direct current stimulation (tDCS)

## Abstract

**Background:**

Binge Drinking (BD) has been associated with altered inhibitory control and augmented alcohol-cue reactivity. Memory inhibition (MI), the ability to voluntarily suppress unwanted thoughts/memories, may lead to forgetting of memories in several psychiatric conditions. However, despite its potential clinical implications, no study to date has explored the MI abilities in populations with substance misuse, such as binge drinkers (BDs).

**Method:**

This study—registered in the NIH Clinical Trials Database (ClinicalTrials.gov identifier: NCT05237414)—aims firstly to examine the behavioral and electroencephalographic (EEG) correlates of MI among college BDs. For this purpose, 45 BDs and 45 age-matched non/low-drinkers (50% female) will be assessed by EEG while performing the Think/No-Think Alcohol task, a paradigm that evaluates alcohol-related MI. Additionally, this work aims to evaluate an alcohol-specific MI intervention protocol using cognitive training (CT) and transcranial direct current stimulation (tDCS) while its effects on behavioral and EEG outcomes are assessed. BDs will be randomly assigned to one MI training group: *combined* [CT and verum tDCS applied over the right dorsolateral prefrontal cortex (DLPFC)], *cognitive* (CT and sham tDCS), or *control* (sham CT and sham tDCS). Training will occur in three consecutive days, in three sessions. MI will be re-assessed in BDs through a post-training EEG assessment. Alcohol use and craving will be measured at the first EEG assessment, and both 10-days and 3-months post-training. In addition, behavioral and EEG data will be collected during the performance of an alcohol cue reactivity (ACR) task, which evaluates attentional bias toward alcoholic stimuli, before, and after the MI training sessions.

**Discussion:**

This study protocol will provide the first behavioral and neurofunctional MI assessment in BDs. Along with poor MI abilities, BDs are expected to show alterations in event-related potentials and functional connectivity patterns associated with MI. Results should also demonstrate the effectiveness of the protocol, with BDs exhibiting an improved capacity to suppress alcohol-related memories after both *combined* and *cognitive* training, along with a reduction in alcohol use and craving in the short/medium-term. Collectively, these findings might have major implications for the understanding and treatment of alcohol misuse.

**Clinical Trial Registration:**

[www.ClinicalTrials.gov], identifier [NCT05237414].

## Introduction

Besides being the most consumed drug in the world, alcohol represents a major risk factor for disease contributing largely to the number of deaths worldwide (World Health Organization [WHO], 2019). WHO data reveals that more than 30% of the deaths of American and European young males aged 15–29 years are somehow associated with alcohol (World Health Organization [WHO], 2011), which suggests that excessive alcohol consumption is particularly harmful for young people. One form of alcohol misuse that is common among youngsters and has received special attention in the last two decades, is binge drinking (BD) which is characterized by episodes of excessive alcohol use followed by periods of low consumption or abstinence (López-Caneda et al., 2019a; Maurage et al., 2020). This pattern is highly prevalent in most Western countries—including Portugal—being present in approximately 35% of people aged 15–24 years (Balsa et al., 2014; Kraus et al., 2016).

The high prevalence of BD at this age is of particular concern since adolescence and youth are periods especially vulnerable to the neurotoxic effects of alcohol, mainly due to the ongoing structural and functional changes occurring in the brain (Jones et al., 2018). Excessive drinking during this neurodevelopmental window might detrimentally influence maturation of cognitive functions, including working memory and/or inhibitory control, relying on still-maturing regions such as the prefrontal cortex (López-Caneda et al., 2014; Lees et al., 2020). Accordingly, BD has been associated with behavioral alterations in verbal memory and executive functions, particularly in inhibitory control and response inhibition to alcohol-specific stimuli (Townshend and Duka, 2005; Czapla et al., 2015; Carbia et al., 2018). Studies using neuroimaging and electroencephalography (EEG) techniques have also revealed abnormalities in the neural correlates of attention and executive functioning related to BD (Crego et al., 2012; Campanella et al., 2013; López-Caneda et al., 2013). Also, evidence from neurofunctional studies showed increased neural reactivity for alcohol-related information in young BDs (Petit et al., 2013, 2014; Brumback et al., 2015; Ryerson et al., 2017; Almeida-Antunes et al., 2022). The altered inhibitory control together with the augmented reactivity to alcoholic stimuli in BDs may constitute a risk factor for the development of alcohol dependence, since it may result in automatic action-tendencies to approach alcohol and difficulties to control alcohol intake (Peeters et al., 2012; Lannoy et al., 2020).

DLPFC has been frequently involved in addictive behaviors, namely in the top-down control processes aiming at regulating motivational reactions to drug cues (Hayashi et al., 2013; Volkow and Morales, 2015). Diminished DLPFC function has been related to reduced cognitive control and higher susceptibility to cue-induced relapse in alcohol abuse (Goldstein and Volkow, 2011). In addition, recent evidence has suggested that the DLPFC plays an important role in memory inhibition (MI), the ability to suppress unwanted or contextually relevant thoughts/memories (Anderson and Hulbert, 2021). MI is commonly studied through the Think/No-Think (TNT) paradigm, an adaptation of the classical Go/NoGo task typically used to evaluate suppression of motor responses (Huster et al., 2013). Briefly, this paradigm is usually divided into three phases (i.e., learning, TNT, and memory-test phases). In the first phase, participants are instructed to learn cue-target pairs, which can be composed of different types of material (i.e., words, pictures, or even autobiographical). During the TNT task, on each trial, a cue from a pair appears in green (Think trials) or red (No-Think trials). For Think trials, participants must remember the paired item and retain it in awareness; for No-Think trials, participants are asked to prevent the paired item from entering awareness. Lastly, the memory-test phase assesses memory for all pairs, with recall measured on Think, No-Think, and Baseline items (i.e., items that were studied during the learning phase but that did not appear in the TNT phase). Studies using this paradigm report consistently two main findings, supporting the assumption that people can voluntarily suppress retrieval (Anderson and Hanslmayr, 2014). Specifically, final recall for No-Think items is significantly reduced than final recall for Think items, suggesting that retrieval suppression reduces the benefits of reminders on memory. Most importantly, suppressing retrieval frequently decreases the memory for No-Think items below that exhibited for Baseline ones, resulting in the suppression-induced forgetting effect or MI (Anderson and Hulbert, 2021).

Growing research has also revealed that forgetting previously learned material involves DLPFC action, which reduces the hippocampus activity and, consequently, impairs memory retrieval (Anderson et al., 2004; Anderson and Hanslmayr, 2014). Recently, López-Caneda and colleagues developed the Think/No-Think Alcohol (TNTA) task, a paradigm aiming to examine the behavioral and neurophysiological mechanisms linked to MI in alcohol-related contexts (López-Caneda et al., 2019b). The authors found lower late parietal positivity (LPP) and increased frontal slow wave (FSW) during No-Think trials, suggesting the involvement of memory suppression mechanisms to drinking (alcoholic and non-alcoholic) contexts (López-Caneda et al., 2019b).

Despite little is known about the MI processes in cases of substance abuse, some studies have demonstrated that alcohol-dependent individuals display anomalies in the ability to intentionally inhibit specific information in comparison with healthy subjects (Noël et al., 2009; Nemeth et al., 2014). Likewise, recent studies have proposed that MI seems to be impaired in several psychiatric disorders, namely in post-traumatic stress disorder (Catarino et al., 2015), attention deficit hyperactivity disorder (Depue et al., 2010) and depressive disorders (Joormann et al., 2009). In the same line, MI training revealed to be effective in enhancing the ability to selectively forget unpleasant memories in both healthy subjects and psychiatric patients (Jacobus and Tapert, 2013; Küpper et al., 2014).

Transcranial direct-current stimulation (tDCS) is one of the most commonly used neuromodulation paradigms in psychological research, which consists of the delivery of a weak electric current from a positive (anode) to a negative (cathode) electrode. It can raise (anodal) or decrease (cathodal) cortical excitability in target regions while also modifying the functional connectivity of the brain networks (Nitsche et al., 2008). This type of stimulation can be paired with cognitive or behavioral interventions to amplify neuroplasticity and boost better longer-term outcomes through synergistic effects (Ditye et al., 2012; Martin et al., 2013). The use of tDCS during multiple intervention sessions has proven to be effective in improving symptoms of several major psychiatric disorders (Kekic et al., 2016) and has recently been suggested as a novel treatment option for substance-use disorders (Ekhtiari et al., 2019). The most frequent anatomical target of these tDCS interventions has been the DLPFC (Boggio et al., 2008) and evidence has shown that tDCS applied over this region may reduce craving and/or alcohol use/relapse in alcohol use disorders (AUD) patients and heavy drinkers (Boggio et al., 2008; da Silva et al., 2013; Jansen et al., 2013; Klauss et al., 2014, 2018; den Uyl et al., 2015; Vanderhasselt et al., 2020). Furthermore, studies applying tDCS over DLPFC coupled with cognitive training (CT) sessions (e.g., cognitive/attentional bias modification; alcohol cue inhibitory control training) also revealed reduced craving in young heavy drinkers (den Uyl et al., 2016), lower relapse rate in recently detoxified patients (den Uyl et al., 2017, 2018; Dubuson et al., 2021) and modifications in the electrophysiological activity of young BDs during the performance of an alcohol-related inhibition task (Dormal et al., 2020). Evidence has shown that DLPFC and hippocampus—two regions specially involved in MI—are classical targets for the neurotoxic effects of alcohol (Jacobus and Tapert, 2013). However, to best of our knowledge, no study has explored the behavioral and neural mechanisms underlying MI neither in alcohol-dependent patients nor in BDs. Thus, based on the evidence that the TNTA paradigm may be a valuable instrument to measure the ability to suppress alcohol-related memories (López-Caneda et al., 2019b), in the present study we will use this task to evaluate the potential electrophysiological and behavioral abnormalities associated with MI, specifically those related to the suppression of alcohol-related memories in young BDs. For this purpose, the electrophysiological activity of 45 college BDs and 45 age-matched non/low-drinkers will be assessed while they perform the TNTA task (pre-training EEG assessment). During this session, psychological (i.e., craving levels), behavioral (i.e., recall accuracy and suppression abilities), and neurofunctional (i.e., Event-Related Potentials [ERPs] and Functional Connectivity [FC]) variables will be assessed. In addition, given that training of response inhibition has been shown to successfully contribute to the reduction –although limited to the short term- of alcohol consumption (Houben et al., 2011), we will develop a coupled tDCS and MI training protocol to investigate whether this training is able (1) to enhance MI capabilities and reduce alcohol cue reactivity (both assessed during the post-training EEG assessment), and (2) to decrease craving and/or alcohol use—monitoring up to 3 months after protocol implementation- in trained BD participants.

## Materials and Methods

### Management and Ethics

Ethical requirements for human research will be followed in full accordance with the Code of Ethical Principles for Medical Research Involving Humans Subjects outlined in the Declaration of Helsinki (64th World Medical Association General Assembly, Brazil, 2013). The Ethics Committee for Social and Human Sciences of University of Minho approved the present protocol in December 12, 2018 (approval reference: CE.CSH 078/2018). Prior to enrolling in the study, subjects will be informed about the aims, conditions and procedure of the study and provided with two copies of the informed consent forms signed by the researchers and participants.. College students will receive gift vouchers in order to compensate for their participation.

### Study Design and Setting

**FIGURE 1:**
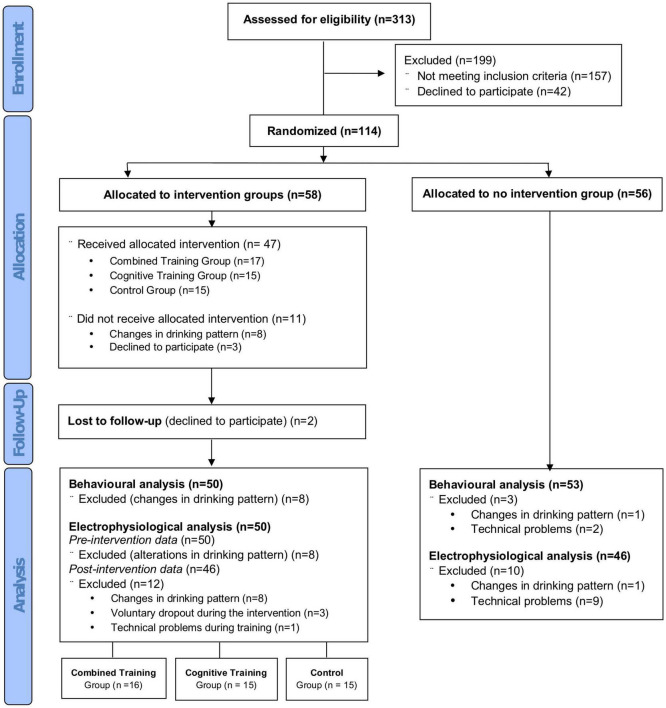
Flow chart of participants recruitment to the study according to CONSORT guidelines.

The authors confirm that all ongoing and related trials of this intervention are registered in the National Institutes of Health (NIH) Clinical Trials Database (ClinicalTrials.gov; identifier: NCT05237414). The CONSORT checklist is available as S1 CONSORT Checklist in Supplementary Material. Participant recruitment started at February 5, 2019 and the anticipated date for follow-up completion is May 15, 2022 (see Figure 1).

**FIGURE 2:**
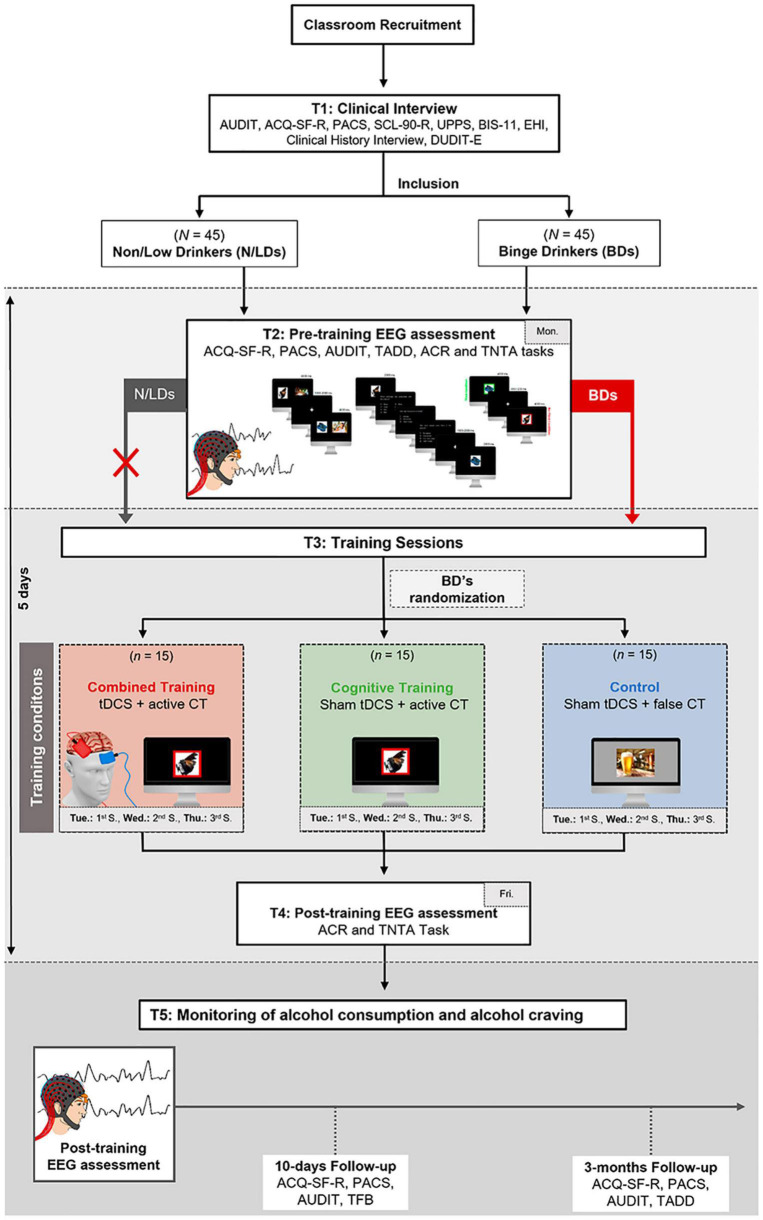
Graphic representation of the procedure. Participants will perform a clinical interview (T1) to guarantee they fulfill the inclusion criteria and to assess baseline measures (e.g., psychological symptoms and impulsivity). Forty-five college students with a BD pattern will enter the study. To compare BDs with non/low-drinkers, the study will also include a group of 45 aged-matched non/low-drinkers, which will only perform the pre-training EEG assessment. During the pre-training EEG assessment (T2; Monday), alcohol craving and consumption levels will be measured, and participants will perform the TNTA task to assess the behavioral and electrophysiological MI mechanisms. Then (T3), BDs will be randomly distributed for one of three training conditions: Combined Training (i.e., verum tDCS and cognitive training [CT]), Cognitive Training (i.e., sham tDCS and verum CT), and Control (i.e., sham tDCS and sham CT). They will perform three sessions over three consecutive days (i.e., Tuesday, Wednesday, and Thursday). After the training sessions, BDs will perform a post-training EEG assessment (T4; Friday) with a procedure similar to T2. The monitoring of alcohol consumption and alcohol craving (T5) will be conducted 10 days and 3 months after T4. ACQ-SF-R, Alcohol Craving Questionnaire-Short Form Revised; ACR, Alcohol Cue Reactivity task; AUDIT, Alcohol Use Disorder Identification Test; BDs, Binge Drinkers; BIS-11, Barratt Impulsivity Scale-11; CT, cognitive training; DUDIT-E, Drug Use Disorders Identification Test-Extended; EEG, electroencephalogram; Fri, Friday; GSI, Global Severity Index; Mon, Monday; N/LDs, Non/low-drinkers; PACS, Penn Alcohol Craving Scale; SCL-90-R, Symptom Checklist-90-Revised questionnaire; TADD, Typical and Atypical Drinking Diary; TLFB, Alcohol Timeline Followback; Tue, Tuesday; Thu, Thursday; UPPS-P, Urgency, Premeditation, Perseverance, Sensation Seeking, Positive Urgency; Wed, Wednesday.

This study is composed of three different phases: (1) behavioral and electrophysiological analyses of MI abilities in young BDs as compared to age-matched non/low-drinkers; (2) a double-blind, randomized controlled trial aiming at assessing the effects of a MI training protocol at the behavioral and electrophysiological level; and (3) monitoring of self-reported alcohol consumption and alcohol craving 10 days and 3 months after the MI training (see Figure 2). Therefore, this experimental procedure aims to answer four main research questions and test specific hypotheses:

1. How does BD affect alcohol-related MI in young adults? Namely, are the behavioral and electrophysiological MI mechanisms—specifically those related to the suppression of alcohol-related memories—altered in BDs when compared to non/low-drinkers? At behavioral level, we hypothesized that BDs would perform worse on the TNTA task, showing an increased recollection of No-Think images, mainly for alcoholic ones, in comparison to N/LDs (i.e., BDs will poorly inhibit alcohol-related pictures). At neurofunctional level, BDs are expected to show alterations in the amplitude of electrophysiological components linked to MI (e.g., N2 and LPP) as well as abnormal FC patterns within/between regions associated with MI (e.g., DLPFC and hippocampal/parahippocampal regions).
2. What is the effect of a MI training on behavioral TNTA task performance? Specifically, will the BDs show a reduced recollection of no-think images—mainly for alcoholic no-think—after training? The results should demonstrate the effectiveness of the training protocol, with BDs exhibiting an improved capacity to suppress alcohol-related memories after both *combined* and *cognitive* MI training.
3. Are the electrophysiological correlates underlying MI mechanisms and alcohol cue reactivity changed by MI training? The MI training protocol should lead to significant modifications in the ERP and FC patterns, reflecting stronger MI capabilities and reduced alcohol cue reactivity in trained BD participants.
4. Will the MI training reduce alcohol consumption and craving levels in the short/medium term? BDs are expected to show a significant reduction in alcohol use and craving in the short/medium-term.

### Target Population

The volunteers will be ninety college students (∼50% female), aged between 18 and 24 years: 45 non/low-drinkers and 45 BDs matched for age and gender. The sample size for the BD group (the group with pre- and post-intervention measures) was determined based on an *a priori* estimation of required sample size with G*Power software (Faul et al., 2009). Parameters for power calculation were: α level = 0.05, effect size = 0.25 (a moderate Cohen’s η^2^; Cohen, 2013), a desired power of 0.90, three manipulation groups (i.e., combined training, cognitive training and control) and three measurements (task performance, and self-reported alcohol use and craving levels). The *a priori* calculation yielded a required sample size of *N* = 45 (i.e., 15 participants in each BD sub-group).). All participants will be recruited through a screening questionnaire administered in the classroom of several courses taught at the University of Minho (UM). The screening will include the Alcohol Use Disorder Identification Test (AUDIT; Babor et al., 2001) along with other questions concerning alcohol and other drugs use.

### Inclusion and Exclusion Criteria

To participate in the study, college students must meet the following eligibility criteria: report (i) drinking 5 or more drinks on one occasion at least once a month, and (ii) drinking at a speed of at least two drinks per hour during these episodes (which brings blood alcohol concentration to 0.08 gram percent or above (National Institute of Alcohol Abuse and Alcoholism [NIAAA], 2004), in order to be classified as BDs; or report (i) never drinking 5 or more drinks on each occasion and (ii) having an AUDIT score ≤ 4, to be considered as non/low-drinkers. The students who fulfilled the inclusion criteria will perform a clinical interview that will assess the following exclusion criteria: (a) use of illegal drugs except cannabis [as determined by the Drug Use Disorders Identification Test-Extended (DUDIT-E), Berman et al., 2007]; (b) alcohol abuse (i.e., AUDIT ≥ 20); consumption of medical drugs with psychoactive effects (e.g., sedatives or anxiolytics) during the 2 weeks before the experiment; (c) personal history of psychopathological disorders (according to DSM-V criteria); (d) history of traumatic brain injury or neurological disorder; (e) family history of alcoholism or diagnosis of other substance abuse; (f) occurrence of one or more episodes of loss of consciousness for more than 20 min; (g) non-corrected sensory deficits; (h) Global Severity Index (GSI) > 90 [Symptom Checklist-90-Revised questionnaire (SCL-90-R), Derogatis, 1983] or a score above 90 in at least two of the symptomatic dimensions.

### Quality Assurance and Randomization

The protocol will be implemented by three skilled researchers with expertise in behavioral and EEG assessments and tDCS interventions. Researchers will not be aware of the results of the pre-training EEG assessment and will be blind to the randomization procedure that will follow. The randomization of the BD groups will be performed by an independent researcher using Microsoft Excel. BDs will receive one of the following interventions: (1) Combined training (CT and verum tDCS applied over the right dorsolateral prefrontal cortex); (2) Cognitive training (active CT and sham tDCS); or (3) Control (sham CT and sham tDCS).

## Experimental Tasks

### Alcohol Cue Reactivity Task

Firstly, the participants will perform the ACR task aiming at assessing the emotional response and the electrophysiological reactivity to alcohol-related cues. In this task, each trial starts with a white fixation cross in a gray background for a variable duration ranging from 1,000 to 1,500 ms. Subsequently, an alcoholic or non-alcoholic image is randomly presented at the center of the screen for 3,000 ms. Participants are asked to be focused on the fixation cross and then to look at the image whenever it appears. After the visualization of each image, participants have to register their emotional responses in terms of valence and arousal using the Self-Assessment Manikin (Bradley and Lang, 1994). The full task includes a total of 80 trials with 40 alcoholic and 40 non-alcoholic images obtained from the Amsterdam Beverage Picture Set (Pronk et al., 2015).

### Think/No-Think Alcohol Task

The TNTA task is a version of the classical Think/No-Think paradigm (Anderson and Green, 2001), which was specially developed to examine MI mechanisms in alcohol-related contexts (López-Caneda et al., 2019b; see Figure 3). This task includes thirty-six pictures (18 related to alcohol and 18 non-related to alcohol) from the Galician Beverage Picture Set (GBPS; López-Caneda and Carbia, 2018) and 36 images of neutral objects obtained from the POPORO database (Kovalenko et al., 2012). The GBPS is a database of alcohol and non-alcohol pictures embedded in real-life scenarios which comprises 6 types of beverages: beer, wine, and liquor (alcoholic drinks), and water, juice, and milk (non-alcoholic drinks). The TNTA task includes 6 images from each of the 6 beverages. The pictures also differ in terms of orientation (vertical or horizontal) and number of people (no people, 1 person, 2 or more people). As such, within each type of beverage, 3 are vertical (each one with a different number of people: 0 people, 1 person, 2 or more people) and the other 3 are horizontal (also with 0 people, 1 person, 2 or more people). The task is divided into three phases, i.e., (1) the Learning phase, (2) the Think/No-Think (TNT) phase and (3) the Memory-Test phase, which are carefully described below.

**Figure:**
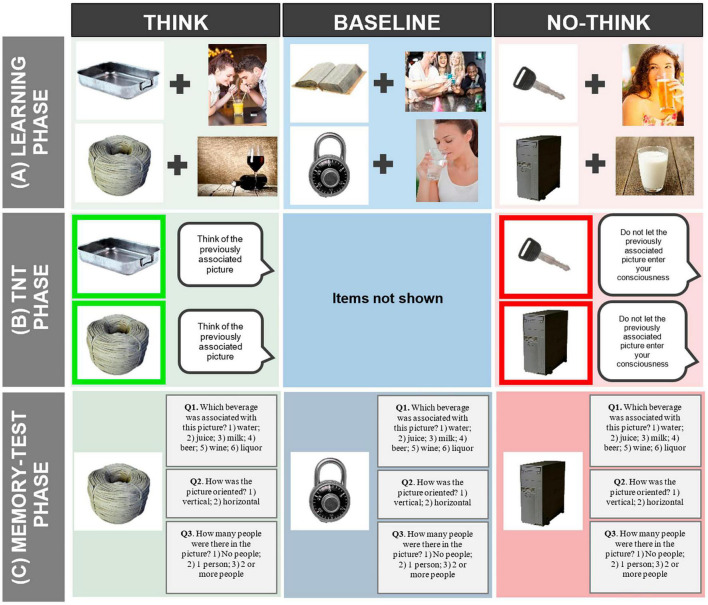
Overall depiction of the Think/No-Think Alcohol (TNTA) task. **(A)** During the learning phase, participants will be asked to associate and memorize 36 pairs of neutral objects + alcoholic/non-alcoholic pictures. Then, only the neutral objects will be presented, and participants will have to try to remember the picture (alcoholic/non-alcoholic image) that was associated with this neutral object and answer three questions about the beverage depicted, the orientation of the picture and the number of people present in it. **(B)** After the learning phase, the Think/No -Think phase will comprehend two conditions: the Think (green square) and the No-Think (light red square). In the Think condition (depicted in the neutral images with a green frame) when participants are presented with the object, they will be instructed to “think of the previously learned alcoholic/non-alcoholic picture and keep it in mind during the entire presentation of the object.” In the No-Think condition (depicted by neutral images with a red frame) they will be asked “not to let the previously associated picture enter your consciousness.” **(C)** In the memory test phase, the 36 neutral images will be presented again, including the 12 neutral objects of the baseline condition that were not presented in the TNT phase. Participants will be asked to recall—answering the same three questions of the learning phase—the image (alcoholic/non-alcoholic) that was initially associated with the neutral object. Alcohol and non-alcohol images were obtained from Shutterstock (https://www.shutterstock.com) and with the permission of Shutterstock.

#### Learning Phase

During the Learning phase (Figure 3A), participants are asked to memorize 36 image-pairs (i.e., a neutral image paired with an image including alcoholic or non-alcoholic drinks) divided into three blocks of 12 pairs. Each block starts with the presentation of the 12 image-pairs at the center of the screen, each for 4,000 ms, in a randomized order, and with an inter-stimuli interval (ISI) ranging from 1,100 to 1,300 ms (with a 4,000 ms rest every 4 pairs). Subsequently, each of the neutral images is presented for 2,000 ms, and participants must try to remember the image that was associated with the neutral object through three questions: Q1. “Which beverage was associated with this picture?”; Q2. “How was the picture oriented?”; Q3. “How many people were there in the picture?” In each block, the 12 pairs and the questions are repeated three times. At the end of each block, feedback with the number of correct responses will be provided. Correct recall will only be considered when participants provide the right answer to the three questions. Thus, the combination of the potential answers to the three questions (6 × 2 × 3 = 36) ensured that each target image displayed a unique combination.

#### Think/No-Think Phase

In this phase (Figure 3B), there are two possible actions: to Think or to No-Think on the alcoholic/non-alcoholic image paired with the neutral object previously. Specifically, in the Think condition—determined by a green frame that circumvents the neutral image -, participants will be asked to focus on the neutral image and think of the alcoholic/non-alcoholic image that was associated with it. In the No-Think condition—neutral image circumvented by a red frame—participants are instructed to focus on the image and not let the previously associated alcoholic/non-alcoholic picture enter their consciousness. Images will randomly repeated 15 times and presented for 3,000 ms (offset-onset ISI = 1,100–1,300 ms). From the initial set of 36 neutral images shown in the learning phase only 24 will be depicted during the TNT phase, 12 in each condition (i.e., Think and No-Think). The remaining 12 neutral images not depicted in this phase will be used as a baseline condition for the following Memory Test phase.

#### Memory Test Phase

During this phase (Figure 3C), the 36 neutral images from each pair are again presented, including the 12 images of the baseline condition. Participants are asked to remember the image (alcoholic or non-alcoholic) that was initially associated with the neutral object using the same three questions of the Learning phase. Three different versions of the task (where all the pictures were part of the three conditions: Think, No-Think and baseline) will be created and counterbalanced across participants.

### Think/No-Think Alcohol Task Variations for Active and False Cognitive Training

Two variations of the TNTA task were developed for the active CT and for the false CT. The stimuli employed in these variations of the TNTA task were also obtained from the GBPS and POPORO databases; however, these stimuli differ from those used in the original TNTA task. The structure of the task for the MI active CT is the same as the original TNTA task. However, in this variation, the Learning phase is only composed of two blocks of 12-image pairs, and in the TNT phase, all the stimuli to be inhibited are alcohol-related images.

In the false CT version, the Learning phase also have only two blocks of 12-image pairs. Nevertheless, the TNT phase is replaced by a Forced-Choice Reaction Time task, during which the participants must only categorize alcoholic and non-alcoholic images answering to the question “What type of beverage was there in the image?” (answer: “Alcoholic drink” or “Non-alcoholic drink”).

All the computerized tasks are programmed in open-source software Psychopy (Peirce, 2007).

## Procedure

### T1: Clinical Interview

All the stages of the procedure are detailed in Figure 2. During the clinical interview (T1), we will verify the fulfillment of the exclusion/inclusion criteria and assess the baseline levels of some constructs (e.g., craving levels, impulsivity). Consequently, the following instruments will be used (see Supplementary Figure 1): (1) the AUDIT along with five additional alcohol use questions (i.e., speed of drinking [number of drinks consumed per hour], number and type of drinks consumed in a standard week, percentage of times getting drunk when going out, age of onset of regular alcohol use and BD); (2) the DUDIT-E to examine other drugs use; (3) the Penn Alcohol Craving Scale (PACS; Pombo et al., 2008) and the Alcohol Craving Questionnaire-Short Form Revised (ACQ-SF-R; Rodrigues et al., 2021); (4) the Barratt Impulsivity Scale-11 (BIS-11; Cruz and Barbosa, 2012) and the short form of the Urgency-Premeditation-Perseverance-Sensation Seeking-Positive Urgency (UPPS-P; Cyders et al., 2014) impulsive behavior scale; (5) the Symptom Checklist-90-Revised questionnaire (SCL-90-R) to evaluate the presence of psychopathological traits; (6) a clinical history interview to explore the personal/family history of psychopathological and neurological disorders as well as the overall medical history of the participants; and (7) the Edinburgh Handedness Inventory (EHI; Espírito-Santo et al., 2017) to evaluate the participants’ handedness.

### T2: Pre-training Electroencephalographic Assessment

During the pre-training EEG assessment (see Figure 2), psychological (i.e., craving levels), behavioral (i.e., alcohol consumption levels and task performance) and neurofunctional (e.g., ERPs, brain FC) outcomes will be assessed (for more details on the instruments used see Supplementary Figure 1). EEG data will be collected while participants perform the ACR and the TNTA task. Before the EEG recording, participants will be asked to perform a breathalyzer test to ensure that blood alcohol concentration is 0.0% (Alcoscan ALC-1). Along with the AUDIT, we will administer the Alcohol Timeline Followback (TLFB) and the Typical and Atypical Drinking Diary (TADD) in order to determine alcohol consumption levels during the previous week and the previous 3 months, respectively. After filling in the questionnaires, participants will perform the ACR task, which lasts from 5 to 10 min. Then, participants’ resting brain activity will be recorded for 3 min during eyes-closed prior to the TNTA task. Finally, they will perform the TNTA task with a duration of 1 h and 10 min. Accordingly, the total duration of the pre-training EEG assessment will be approximately two and a half hours.

EEG data will be recorded using the ActiveTwo Biosemi System (Biosemi, Inc.) from 64 electrodes placed according the 10–10 system (Fregni et al., 2015) and digitized at a 512 Hz rate. Vertical and horizontal electrooculogram activity will be recorded to control for eye movements and blinks. Two additional electrodes will be placed on the mastoids, bilaterally, to provide the signal offline reference. Electrode impedances will be kept below 20 kΩ and the EEG signal will be filtered on-line with a 0.01–100 Hz band pass filter.

### T3: MI Training Sessions

BDs will be randomly assigned to one of the three training subgroups: (a) *Combined training*; (b) *Cognitive training*; or (c) *Control* (see Figure 2). The participants’ allocation to the training group will be done by an independent researcher who will be responsible to program the tDCS parameters. Thus, both participants and research team will be blind to the randomization procedure.

During T3, subjects will perform the variation of the TNTA task corresponding to the group to which they will be assigned. After the first phase of the task (i.e., Learning Phase), neuromodulation (sham or active) will be performed using tDCS. Twenty minutes of 2 mA direct current will be applied to the scalp using a saline-soaked pair of 35 cm^2^ surface sponge electrodes, through an Eldith DC Stimulator Plus (Neuroconn, Germany). To stimulate the right DLPFC, the anodal electrode will be placed over F4 according to the 10–20 international system for EEG electrode placement. The cathode electrode will be placed over the contralateral supraorbital area. During the active simulation, the current will fade in for 15 s, will be constant at 2 mA for 20 min, and then will fade out for 15 s. During the sham stimulation, the electric current will fade in for 15 s, then will be constant at 2 mA for 15 s and will fade out for 15 s. This procedure makes both active and sham stimulation indistinguishable for the participants. Before and after the stimulation, participants will answer to a continuous Visual Analog Scale that allows checking for possible secondary effects of the electrical stimulation.

### T4: Post-training Electroencephalographic Assessment

During T4 (see Figure 2), participants will perform the ACR and TNTA tasks under the same procedure to the one undertaken during the T2.

### T5: Monitoring of Alcohol Consumption and Alcohol Craving

Ten days after the T4, the primary craving and alcohol consumption outcomes will be assessed (see Figure 2). For this purpose, we will administer the PACS, the ACQ-SF-R and the AUDIT. Additionally, 3 months post-intervention, BDs will answer the same questionnaires and also the TADD aiming at assessing potential medium-term effects of the MI training.

## Data Collection and Analysis

### Data Collection

Data collection will occur, firstly, at university classrooms at the UM during the screening phase, and afterward at the facilities of the Psychological Neurosciences Laboratory at the School of Psychology of UM (Braga, Portugal). Participants will answer the questionnaires by using paper and pencil, and the data will then be entered into SPSS^®^ (Version 27.0). Behavioral data obtained from the experimental tasks will be collected with PsychoPy (v1.84.0).

### Electroencephalographic Data Processing

EEG data will be processed with FieldTrip package (Oostenveld et al., 2011). The signal will be corrected for vertical and horizontal ocular artifacts by independent component analysis (ICA) and re-referenced to the average reference For each subject, the normal distribution of raw signal (restricting its frequency range using band-pass filter for each type of artifact) will be calculated. Signals ≥ 2 standard deviations from mean score will be automatically marked as artifacts. Then, the correct artifacts removal will be visually inspected by an expert signal analyst. Therefore, every trial affected by movement or other kind of artifacts will be discarded from subsequent analysis. The data will be segmented into epochs of 2,500 ms (from −500 to 2,000 ms after stimulus onset) and trials marked as containing artifacts will be discarded from subsequent analysis. Only trials corresponding to originally learned items during the learning phase will be considered. Additionally, these epochs will be averaged separately according to the type of picture to be recalled or suppressed, thus obtaining four conditions: Alcohol Think, No-Alcohol Think, Alcohol No-Think and No-Alcohol No-Think.

#### Event-Related Potentials Analysis

To quantify the ERP data, we will calculate the mean amplitudes for each electrode in several time-windows, selected based on previous findings (e.g., López-Caneda et al., 2019b) and on the visual inspection of the ERP waveforms (predictably, 200–400 ms and 600–1,000 ms for fronto-central locations and 400–700 ms for parietal locations). We will use this selection in order to quantify the N2, FSW, and LPP components: to explore the N2 and the FSW amplitudes, we will extract the ERP data from six electrodes placed at left (F3, FC3), midline (Fz, FCz), and right (F4, FC4) frontal regions and to study the LPP component, the ERP data will be extracted from the following scalp electrodes: left parietal (P3, PO3), midline parietal (Pz, POz), and right parietal (P4, PO4).

#### Functional Connectivity Analysis

EEG data will be transformed into source space employing a realistic *Boundary elements model* (BEM) (Fuchs et al., 2001) as forward model and a Linearly Constrain, Minimum Variance (LCMV) beamformer (Van Veen et al., 1997) as inverse model. Cortical sources will be reconstructed in five classical bands—theta (4–8 Hz), alpha (8–12 Hz), low beta (12–20 Hz), high beta (20–30 Hz) and low gamma (30–45 Hz)-, using the Montreal Neurological Institute (MNI) template-based T1 images (ICBM 152) (Mazziotta et al., 2001). FC analysis will be calculated under the hypothesis of phase synchronization by means of the Phase Locking Value (PLV) (Bruña et al., 2018).

### Statistics

The first step of the statistical analyses will be to examine the behavioral and EEG correlates of MI among college BDs and non/low-drinkers. For that purpose, the outcomes of the TNTA task collected during the pre-training assessment (T2) in BDs and non/low-drinkers will be compared and correlated with alcohol consumption (AUDIT) and alcohol craving measures (i.e., the PACS’ and ACQ-SF-R’s scores, and the ERPs and valence/arousal ratings recorded during the ACR task). Secondly, with the purpose of verifying the impact of the *combined, cognitive* and *control* training procedures applied to BDs on psychological, behavioral, and neurofunctional measures, the pre-training data (T2) from the 45 BDs will be compared with data from T4 (post-training) and T5 (monitoring of alcohol consumption and alcohol craving). In addition, the three training groups will be compared with each other in order to determine potential differences between the training procedures at T4 and T5 stages.

With regard to the behavioral data, items learned during the learning phase and correctly recalled during the memory test phase will be considered correct responses. Accordingly, the percentage of correct responses (for Think, No-Think and Baseline items) will be computed according to the following formula:


$$\left(\frac{n\,u\,m\,b\,e\,r\,o\,f\,c\,o\,r\,r\,e\,c\,t\,l\,y\,r\,e\,c\,a\,l\,l\,e\,d\,i\,t\,e\,m\,s}{n\,u\,m\,b\,e\,r\,o\,f\,p\,r\,e\,v\,i\,o\,u\,s\,l\,y\,l\,e\,a\,r\,n\,e\,d\,i\,t\,e\,m\,s}\right)\times 100$$

A mixed-model analysis of variance (ANOVA) with one between-subject factor, Group (non/low-drinkers, BDs), and two within-subject factors, Condition (Think, No-Think, Baseline) and Content (Alcohol, Non-Alcohol) will be conducted on the recall accuracy rate to examine the participants’ MI ability at T2. Afterward, a repeated-measures ANOVA with three within-factors: Moment (T2 and T4), Condition (Think, No-Think, Baseline) and Content (Alcohol, Non-Alcohol) will be performed to explore the training effects on the MI ability of BDs.

Furthermore, to examine the participant’s emotional response to alcoholic cues (self-assessed during the ACR task using the Manikin test) at T2, two ANOVAs with Group (non/low-drinkers, BDs) as between-subject factor and with Content (Alcohol, Non-Alcohol) as within-subject factor will be conducted for valence and arousal ratings, separately. In addition, to evaluate possible variations in valence and arousal responses as a function of training sessions, new ANOVAs will be performed for each training group with two within-factors—Moment (T2 and T4) and Content (Alcohol, Non-Alcohol).

For the TNTA task, we will analyze the ERPs, specifically the mean amplitudes of N2, LPP, and FSW. A mixed-model ANOVA with one between-subject factor Group (non/low-drinkers, BDs) and four within-subject factors: Condition (Think, No-Think), Content (Alcohol, Non-Alcohol), Region (Left, Midline, Right), and Electrode (2 electrodes) will be conducted on the mean amplitude of each component to explore the MI neural mechanisms during T2. A repeated-measures ANOVA with five within-subject factors: Moment (T2 and T4), Condition (Think, No-Think), Content (Alcohol, Non-Alcohol), Region (Left, Midline, Right) and Electrode (two electrodes) will be conducted on the mean amplitude of each component separately, to explore the effects of MI training on the neural activity.

For the ACR task at T2, the mean amplitude of the P1, N1, and P2 ERP components will be analyzed by means of separate mixed-model ANOVAs with one between-subject factor Group (non/low-drinkers, BDs) and three within-subject factors: Content (Alcoholic, Non-Alcoholic), Region (Left, Midline, Right) and Electrode (2 electrodes). Furthermore, in order to investigate potential MI training effects on the electrophysiological reactivity to alcoholic cues, the amplitude of the abovementioned components will be analyzed through repeated-measures ANOVAs using within-subject factors: Moment (T2 and T4), Content (Alcoholic, Non-Alcoholic), Region (Left, Midline, Right) and Electrode (two electrodes). The behavioral and ERP correlates of MI and alcohol craving/reactivity will be correlated with the scores obtained from AUDIT, ACQ-SF-R, and PACS.

Regarding FC assessment, for the nodal strength—i.e., the level of connectivity of each node with the rest of the network-, the data will consist of a single value per source. These values will be compared between groups using a cluster-based permutation test (CBPT) (Oostenveld et al., 2011). For the seed-based analysis, and according to the literature concerning MI, prefrontal (e.g., DLPFC) and medial temporal (e.g., perirhinal and parahippocampal cortex, hippocampus) regions will be used as seeds. Lastly, the regions defined by the clusters showing significant differences between both groups in the nodal strength analysis will also be used as seeds.

## Discussion

At the present, the literature is still scarce regarding both the core functional anomalies in BDs and the neurobiological factors that underlie the evolution from pre-clinical (e.g., hazardous alcohol use, BD) to clinical conditions related to alcohol (i.e., AUD) (Witkiewitz et al., 2019). This study protocol constitutes an important step to fill in the existing gap and an opportunity to shed new light on a broad and more comprehensive understanding of the memory suppression mechanisms and their potential implication on heavy alcohol use. To the best of our knowledge, this protocol will be the first to describe the design and implementation of a randomized controlled trial that examines MI in BDs and tests the effectiveness of different types of MI training using both CT and neuromodulation by tDCS with the aim of reducing alcohol use and craving. Examining the extent to which BDs may have difficulties to inhibit alcohol-related information, what its behavioral and neurophysiological underpinnings are and how they can be potentially modified by training, will extend previous research on inhibition training in social and problem drinkers (Petit et al., 2012; Jones et al., 2014; Di Lemma and Field, 2017; Smith et al., 2017; Kilwein et al., 2018). Moreover, findings resulting from this research could hold important clinical implications for alcohol misuse treatment, particularly for that focused on the training of response inhibition to alcohol cues (Houben et al., 2011, 2012; Tschuemperlin et al., 2019).

Besides its innovative character granted by the use of a neuromodulation technique and the collection of behavioral and electrophysiological data, the protocol involves the longitudinal assessment of important psychological variables and measurements of alcohol craving/consumption registered at follow-up periods of up to 3 months. This monitoring will allow a comprehensive proximal and distal characterization of potential changes in BD behavior resulting from the MI training.

Ultimately, the present study may be particularly important as it can provide a new and useful tool for the clinical community and, consequently, contribute to improving the quality of life of individuals suffering from problems related to alcohol and/or other substances. Moreover, our findings might encourage other researchers to conduct new studies on this topic and, thus, lead to a build-up of a strong and more comprehensive body of knowledge involving MI in substance misuse that can be translated into measurable societal impact.

## Ethics Statement

This study, involving human participants, was reviewed and approved by Ethics Committee for Social and Human Sciences of University of Minho (approval reference: CE.CSH 078/2018). The participants provided their written informed consent to participate in this study.

## Author Contributions

EL-C: conceptualization. EL-C, AC, and AS: funding acquisition and methodology. EL-C and AC: project administration. EL-C, AC, AS, NA-A, MV, and RR: validation. NA-A and MV: visualization and writing – original draft. EL-C, AC, AS, and RR: writing – review and editing. All authors contributed to the article and approved the submitted version.

## Conflict of Interest

The authors declare that the research was conducted in the absence of any commercial or financial relationships that could be construed as a potential conflict of interest.

## Publisher’s Note

All claims expressed in this article are solely those of the authors and do not necessarily represent those of their affiliated organizations, or those of the publisher, the editors and the reviewers. Any product that may be evaluated in this article, or claim that may be made by its manufacturer, is not guaranteed or endorsed by the publisher.
