# Supplementary material for: Forgetting Alcohol: A Double-Blind, Randomized Controlled Trial Investigating Memory Inhibition Training in Young Binge Drinkers
Source: Front Neurosci. 2022 Jun 29;16:914213. doi: 10.3389/fnins.2022.914213 (PMC9278062; doi:10.3389/fnins.2022.914213)
Supplement: Supplementary file 1 [file Image_1.pdf]

Supplementary material

	T1: Clinical History Interview	T2: Pre-training EEG assessment	T3: Training Sessions	T4: Post-training EEG assessment	T5.1: 10-days follow-up	T5. 2: 3-months follow-up
**Alcohol and other Substance use**	AUDIT<br>**+**<br>**5 alcohol use questions**:<br>• Speed of Drinking<br>• Consumption in a standard week<br>• Percentage of times getting drunk<br>• Age of onset of regular drinking<br>• Age of onset of BD<br>DUDIT-E	AUDIT<br>**+**<br>**1 alcohol use question**:<br>• Speed of Drinking<br>TLFB<br>DUDIT-E			AUDIT<br>**+**<br>**1 alcohol use question**:<br>• Speed of Drinking<br>TLFB	AUDIT<br>**+**<br>**1 alcohol use question**:<br>• Speed of Drinking<br>TLFB<br>TADD
**Alcohol Craving**	ACQ-SF-R<br>PACS	ACQ-SF-R<br>PACS			ACQ-SF-R<br>PACS	ACQ-SF-R<br>PACS
**Other**	EHI<br>UPPS-P<br>BIS-11<br>SCL-90-R	Breathalyzer test<br>EEG Session Checklist<br>tDCS Screening Questionnaire	Breathalyzer test<br>VAS	Breathalyzer test<br>EEG Session Checklist		
**Computerized Tasks**		ACR Task<br>TNTA Task	Variations of the TNTA task	ACR Task<br>TNTA Task		

S1 Fig 1. Table describing the questionnaires and tasks administered throughout the different steps of protocol. Note: Note: ACQ-SF-R - Alcohol Craving Questionnaire-Short Form Revised; ACR – Alcohol Cue Reactivity task; AUDIT - Alcohol Use Disorder Identification Test; BDs – Binge Drinkers; BIS-11 - Barratt Impulsivity Scale-11; CT – cognitive training; DUDIT-E - Drug Use Disorders Identification Test-Extended; EEG – electroencephalogram; GSI - Global Severity Index; N/LDs – Non-/Low Drinkers; PACS – Penn Alcohol Craving Scale; SCL-90-R - Symptom Checklist-90-Revised questionnaire; TADD - Typical and Atypical Drinking Diary; TLFB - Alcohol Timeline Followback; UPPS-P - Urgency-Premeditation-Perseverance-Sensation Seeking-Positive Urgency.
